# Immunohistochemical evaluation of T cell receptor and T cell receptor beta constant 1 expression distinguishes benign and neoplastic immature T-cell populations and reveals discrete TRBC1/TCR phenotypes

**DOI:** 10.1007/s12308-025-00674-2

**Published:** 2025-12-13

**Authors:** Sahil Chaudhary, Elizabeth L. Courville, Jeffrey Craig, Vanessa Smith, Margaret Moore

**Affiliations:** Department of Pathology and Laboratory Medicine, University of Virginia Health, 1215 Lee Street, Charlottesville, VA 22908 USA

**Keywords:** TRBC1, Thymus, Thymoma, T-lymphoblastic leukemia/lymphoma, Immunohistochemistry, Clonality

## Abstract

**Background:**

Expression of TRBC1 and TRBC2 is increasingly assessed in the evaluation for clonal T-cell populations. While flow cytometry has repeatedly shown utility in evaluating TRBC 1/2 expression, immunohistochemical (IHC) methods have not been extensively examined, particularly in immature T-cell populations.

**Purpose:**

This study evaluated TRBC1 IHC staining in formalin-fixed paraffin-embedded (FFPE) tissue, encompassing benign thymic tissue, thymomas, and T-lymphoblastic leukemias/lymphomas (T-LL).

**Methods:**

IHC for TRBC1, CD3, TCR-BF1, and TCR-δ was performed in all cases; TdT was performed on all T-LLs. TRBC1 was scored as positively restricted (≥ 85%), negatively restricted (≤ 15%), or polytypic (16–84%) in T cells, using CD3 and/or TdT staining as the denominator.

**Results:**

All thymic tissues (*n* = 6) and thymomas (*n* = 5) showed polytypic TRBC1 staining patterns. Twenty-four T-LL specimens were identified from 21 patients, including bone marrow (*n* = 16), lymph node (*n* = 7), and bone (*n* = 1) biopsies. All T-LL cases showed positively (*n* = 3 patients, 14%) or negatively (*n* = 18 patients, 86%) restricted TRBC1 expression. Patients with multiple specimens showed consistent TRBC1 staining across tissue sites and sampling time points. Coexpression analysis of TRBC1, TCR-BF1, and TCR-δ staining revealed frequent TRBC1-negative cases with either TCR-δ + (*n* = 6, 29%) or TCR null (*n* = 7, 33%) phenotypes. TRBC1 IHC restriction and molecular methods for clonality assessment were concordant in 13 of 15 evaluated cases (87%).

**Conclusions:**

T-LL showed restricted patterns of TRBC1 expression by IHC, whereas benign immature T-cell populations showed polytypic staining. The observed TCR and TRBC1 phenotypes of T-LL are distinct from those of mature T-cell neoplasms and warrant further study.

**Supplementary information:**

The online version contains supplementary material available at 10.1007/s12308-025-00674-2.

## Introduction

The distinction between neoplastic and non-neoplastic T-cell proliferations can be challenging, and assessment of T-cell clonality assists in accurate diagnostic classification. Genetic-based strategies such as polymerase chain reaction (PCR) and next-generation sequencing (NGS) analyze T-cell receptor (TCR) gene rearrangement profiles to identify clonal T-cell proliferations [1]. Previous phenotype-based flow cytometry assays utilized large panels of antibodies targeting families of TCR beta-chain variable regions [2]. Evaluation of TCR beta constant region (TRBC) expression is a recently developed strategy to assess T-cell clonality [3, 4]. As a consequence of functional TCR rearrangement, a TCR α/β-positive T cell expresses either TRBC1 or TRBC2 as part of its TCR [5]. Polyclonal TCR α/β T-cell populations therefore contain a mixture of cells expressing TRBC1 or TRBC2, whereas clonal populations demonstrate “restricted” expression of one TRBC isoform [6]. A small subset of TCR α/β-derived T-cell neoplasms show a “TRBC null” phenotype, lacking expression of both TRBC1 and TRBC2 [7].

Flow cytometry is a robust method for assessing TRBC expression, with well-established utility in identifying clonal T-cell populations [6–11]. However, flow cytometry is not readily available for all sample types, nor in all practice settings. Immunohistochemistry (IHC) represents an alternative method to evaluate TRBC expression that can be performed on formalin-fixed, paraffin-embedded (FFPE) tissues. In early studies, TRBC IHC showed utility in differentiating between non-neoplastic and neoplastic mature T-cell processes, demonstrating a high level of concordance with clonality assessment by flow cytometry and molecular methods [12–14]. IHC allows for spatial evaluation of TRBC staining in the context of other morphologic features and allows for staining assessment in both membranous and cytoplasmic cellular compartments. Flow cytometry assays are commonly optimized to evaluate surface TRBC expression and require additional processing steps to measure cytoplasmic expression (as when analyzing surface CD3-negative T-cell populations) [11, 15].

In this study, we analyzed TRBC1 IHC staining in a cohort of benign thymic tissue, thymoma, and T-lymphoblastic leukemia/lymphoma (T-LL). Our primary aims were to contribute to the emerging body of literature describing TRBC1 staining in FFPE tissue samples and describe our experience in assessing TRBC1 expression in immature T-cell populations, which have not been well represented in earlier publications.

## Materials and methods

This study was approved by our institutional review board (HSR #13310).

### Sample selection

A retrospective search of pathology department archives was performed (2019–2024) to identify cases of T-LL, not otherwise specified, and early T-precursor lymphoblastic leukemia/lymphoma (ETP-LL) with available formalin-fixed, paraffin-embedded (FFPE) tissue. T-LL cases were required to meet diagnostic criteria per the fifth edition of the World Health Organization Classification of Haematolymphoid Tumors [16]. Tissue biopsies were required to show architectural effacement by a diffuse proliferation of T-lymphoblasts. Bone marrow specimens were required to have at least 20% blasts. Representative excisional specimens of benign thymic tissue and thymomas (types AB, B1, and B2) were chosen to represent benign populations of immature T cells. Diagnoses for all cases were confirmed by a board-certified hematopathologist.

### Immunohistochemistry

For all cases (benign thymic tissue, thymoma, T-LL), an H&E slide and IHC stains for CD3 (Leica Biosystems, LN10), TCR-BF1 (Thermo Fisher Scientific, 8A3), TCR-δ (Santa Cruz, H-41), and TRBC1 (Cell Signaling Technology, E6Z3S, 1:200 dilution) were performed. In addition, pan-cytokeratin (institutional keratin cocktail) was performed on all thymoma cases, and TdT (Leica, SEN28) was performed on all T-LL cases. Additional IHC stains performed on T-LL cases as part of the original diagnostic evaluation were also reviewed, as available, including CD2 (Leica, 11F11), CD5 (Leica, 4C7), CD7 (Leica, LP15), CD4 (Leica, 4B12), CD8 (Leica, 4B11), CD1a (Leica, MTB1), CD34 (Leica, QBEnd/10), CD10 (Leica, 56C6), CD123 (BD, 7G3), CD117 (Leica, EP10), CD99 (CM, EPR3097Y), CD56 (Leica, CD564), and/or CD38 (Epitomics, EP-135).

IHC stains were manually interpreted in the T-cell populations of interest using conventional light microscopy. All stains were independently scored by two board-certified hematopathologists, each blinded to the other’s interpretation. Any stain lacking a consensus interpretation was independently reviewed by a third pathologist, with the majority opinion (agreement between two of three pathologists) being adopted as the final phenotype.

TRBC1 was semi-quantitatively scored in all samples as “negatively restricted” if ≤ 15% of the T-lineage population (thymocytes/T-lymphoblasts) showed expression, “positively restricted” if ≥ 85% showed expression, and “polytypic” if 16–84% showed expression. These thresholds were selected based on proposed TRBC1% cutoffs in existing flow cytometry literature [8–11]. In assessing the proportion of TRBC1 + cells, pathologists scored TRBC1 in comparison to CD3 (TRBC1/CD3%) and TdT (TRBC1/TdT%, available for all cases of T-LL), utilizing discretion to determine which marker best delineated the immature T-cell population of interest.

For cases of T-LL, all remaining IHC stains were interpreted as “positive” or “negative” in the T-lymphoblast population, with reviewing pathologists providing additional descriptors of staining quality (weak, subset, variable, etc.), if desired.

### Flow cytometry

Clinical-grade multiparametric flow cytometry was performed on T-LL cases at the time of original diagnostic evaluation. None of the included cases of benign thymic tissue or thymoma had flow cytometry performed. Flow cytometry data was retrospectively reviewed, with the phenotype of the T-lymphoblast population recorded for each case. Cases from the study period were analyzed by either six-color flow cytometry panels on FACSCanto instruments (2019–2024) or ten-color panels on FACSLyric instruments (adopted at the institution mid-2024). Positive and negative staining for the markers of interest was established by comparison of the blast profile to internal control populations of known phenotypes.

In particular, surface expression of TRBC1 (Jovi-1, FITC-conjugated) was assessed as part of the following monoclonal antibody cocktail: TRBC1/TCRγ/δ/CD5/CD3/CD8/CD7/CD2/CD4/CD45. TRBC1 expression in ≥ 85% or ≤ 15% of the population of interest was reported as positively or negatively restricted, respectively.

### TCR gamma gene rearrangement

DNA was extracted from fresh or formalin-fixed paraffin-embedded samples using a modified version of the QIAGEN QIAamp DNA purification protocol. The master mix containing the multiple overlapping primer sets utilized for PCR of the TCRγ chain genes was developed by InVivoScribe Technologies. Following PCR, amplicon analysis was performed by capillary electrophoresis on the ABI 3500XL instrument. The assay is able to detect a 2–3% clonal population in a polyclonal background under ideal conditions. All TCR PCR studies were reviewed by a board-certified molecular pathologist.

### ClonoSEQ

ClonoSEQ T-cell clonality assessment was performed at the time of diagnosis on select cases (Adaptive Biotechnologies, proprietary), and available results were extracted from the electronic medical record.

## Results

### Immunohistochemistry

#### Benign thymic tissue and thymoma

Six representative excisional specimens containing benign thymic tissue were selected, including biopsies from the thymus (*n* = 5), as well as ectopic thymic tissue associated with a parathyroid gland (*n* = 1). Five representative thymoma excisions were also analyzed, including types B1 (*n* = 2), B2 (*n* = 2), and AB (*n* = 1).

**Fig. 1:**
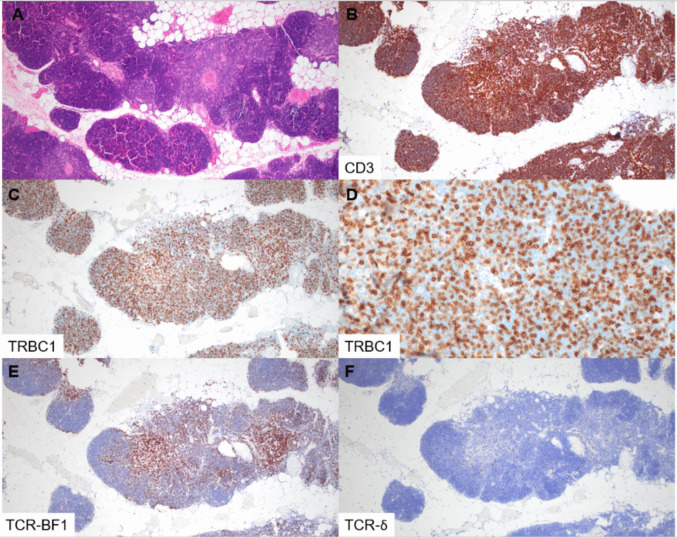
Staining profile of TRBC1 in benign thymus. Benign thymic tissue (**A**, H&E) showing a predominance of CD3-positive T-lineage cells (**B**). TRBC1 (**C**, **D**) is positive in only a subset of the T cells, compatible with a polytypic (16–84%) staining profile. TCR-BF1 shows staining in many of the T cells (**E**), with dim staining within the cortical region and more intense staining in the medullary region. TCR-δ (**F**) is positive in only sparse, scattered cells (magnification 100×, **A**, **B**, **C**, **E**, **F**; 400×, **D**)

All cases of benign thymic tissue and thymoma showed an expected predominance of CD3-positive T-lineage cells within the lymphoid compartment (Fig. 1). The vast majority of T cells expressed TCR-BF1, with more intense staining in the medullary areas, while only a minority of T cells expressed TCR-δ. For all cases of thymic tissue and thymoma, TRBC1 expression was scored as polytypic in the T- cells of interest by both reviewing pathologists.

#### T-lymphoblastic leukemia/lymphoma

Twenty-four cases of T-LL were identified, from 21 unique patients (Table 1), including 16 bone marrow biopsies, seven lymph node specimens, and one targeted biopsy of a bone lesion. Twenty-two samples represented the initial, diagnostic specimen, while two specimens were collected in the setting of disease relapse.

**Table 1:** T-lymphoblastic leukemia/lymphoma patient demographics

Patient #	Specimen #	Sex	Age (years)	Tissue type	Clinical history
1	1	M	20	Bone marrow, clot preparation	Leukocytosis and mediastinal mass
2	2	M	24	Lymph node, core biopsy	Initial presentation with fatigue, cervical lymphadenopathy, and mediastinal mass
2	3	M	24	Bone marrow, clot preparation	Disease relapse after incomplete chemotherapy protocol
3	4	F	48	Bone marrow, clot preparation	Bilateral cervical lymphadenopathy
4	5	F	6	Lymph node, excision	Pleural effusion, hepatomegaly and lymphadenopathy
5	6	M	43	Bone marrow, core biopsy and clot preparation	Bilateral pleural effusion and anterior mediastinal mass
6	7	M	55	Bone marrow, core biopsy	History of AML*, status post allogeneic stem cell transplantation, presenting with pancytopenia and circulating blasts
7	8	M	39	Bone marrow, core biopsy	Leukocytosis, lymphadenopathy, and hepatosplenomegaly
8	9	M	14	Bone marrow, core biopsy	Leukocytosis and cervical lymphadenopathy
8	10	M	14	Lymph node, excision	
9	11	M	28	Bone marrow, core biopsy	Fatigue, circulating atypical lymphocytes on peripheral smear review
10	12	M	27	Bone lesion (targeted), core biopsy	Metabolically active bone lesion detected on surveillance
11	13	M	45	Bone marrow, clot preparation	Disease relapse
12	14	M	2	Bone marrow, core biopsy	Bilateral cervical adenopathy
12	15	M	2	Lymph node, excision	
13	16	M	7	Bone marrow, core biopsy, and clot preparation	Leukocytosis, hepatosplenomegaly, abdominal pain
14	17	M	36	Bone marrow, core biopsy, and clot preparation	Pleural effusion and mediastinal mass
15	18	M	38	Bone marrow, core biopsy	Leukocytosis, pleural effusion, mediastinal mass
16	19	M	18	Lymph node, excision	Mediastinal mass and diffuse lymphadenopathy
17	20	M	53	Lymph node, core biopsy	Bilateral inguinal lymphadenopathy and scrotal swelling
18	21	M	6	Bone marrow, core biopsy, and clot preparation	Mediastinal mass, abdominal distension, features of tumor lysis syndrome
19	22	M	22	Bone marrow, core biopsy, and clot preparation	Pleural effusion, mediastinal mass, and lymphadenopathy
20	23	M	43	Lymph node, excision	Multistation lymphadenopathy
21	24	M	4	Bone marrow, core biopsy	Hypercalcemia, anemia, thrombocytopenia

^*^AML acute myeloid leukemia. The T-lymphoblast population was immunophenotypically distinct from the patient’s reported myeloid neoplasm and had no features of a mixed phenotype leukemia

**Fig. 2:**
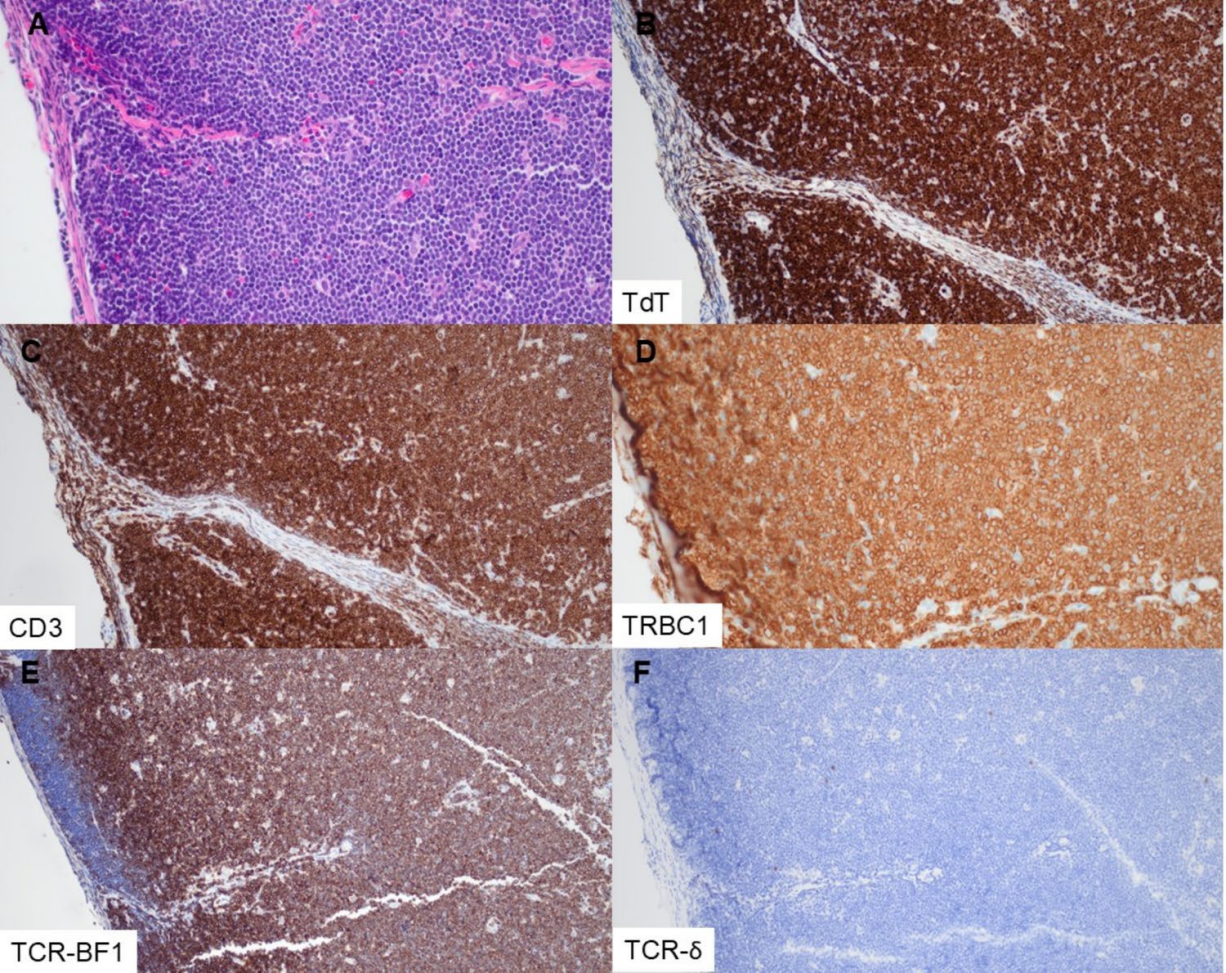
T-LL with positively restricted TRBC1 expression. This lymph node (**A**, H&E) from patient 12, specimen 15, shows extensive involvement by the T-lymphoblast population and exhibits uniform expression of TdT (**B**) and CD3 (**C**). TRBC1 is diffusely positive in the cells of interest (**D**), compatible with positive restriction. The neoplastic cells are positive for TCR-BF1 (**E**) and lack expression of TCR-δ (**F**) (magnification 200×, all panels)

**Fig. 3:**
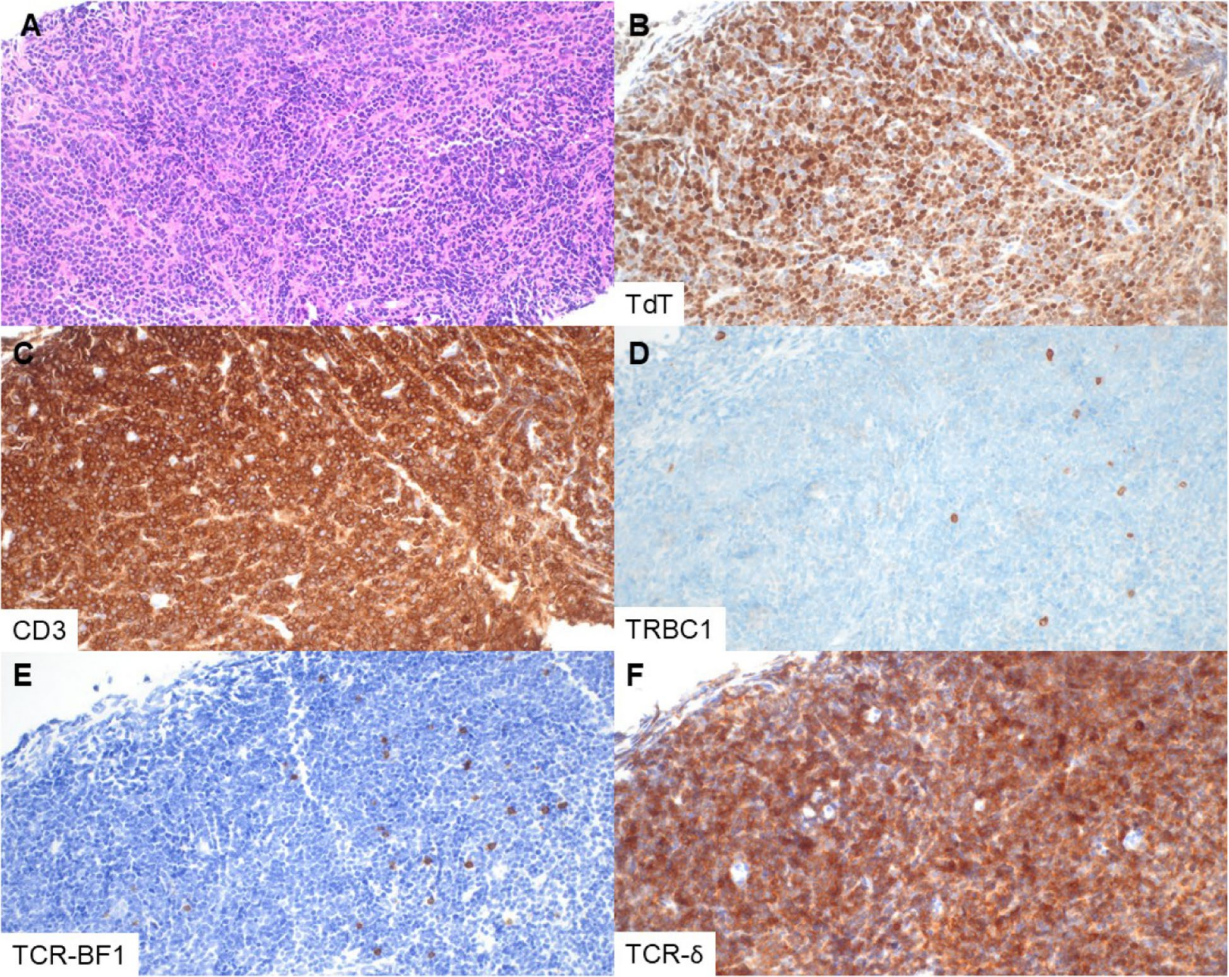
T-LL with negatively restricted TRBC1 expression and strong expression of TCR-δ. This lymph node core biopsy (patient 2, specimen 2) is effaced by sheets of lymphoblasts, seen by H&E (**A**), TdT (**B**), and CD3 (**C**). TRBC1 is uniformly negative in the cells of interest (**D**), but highlights sparse background non-neoplastic T cells. The neoplastic cells are negative for TCR-BF1 (**E**) and strongly positive for TCR-δ (**F**) (magnification 400×, all panels)

**Fig. 4:**
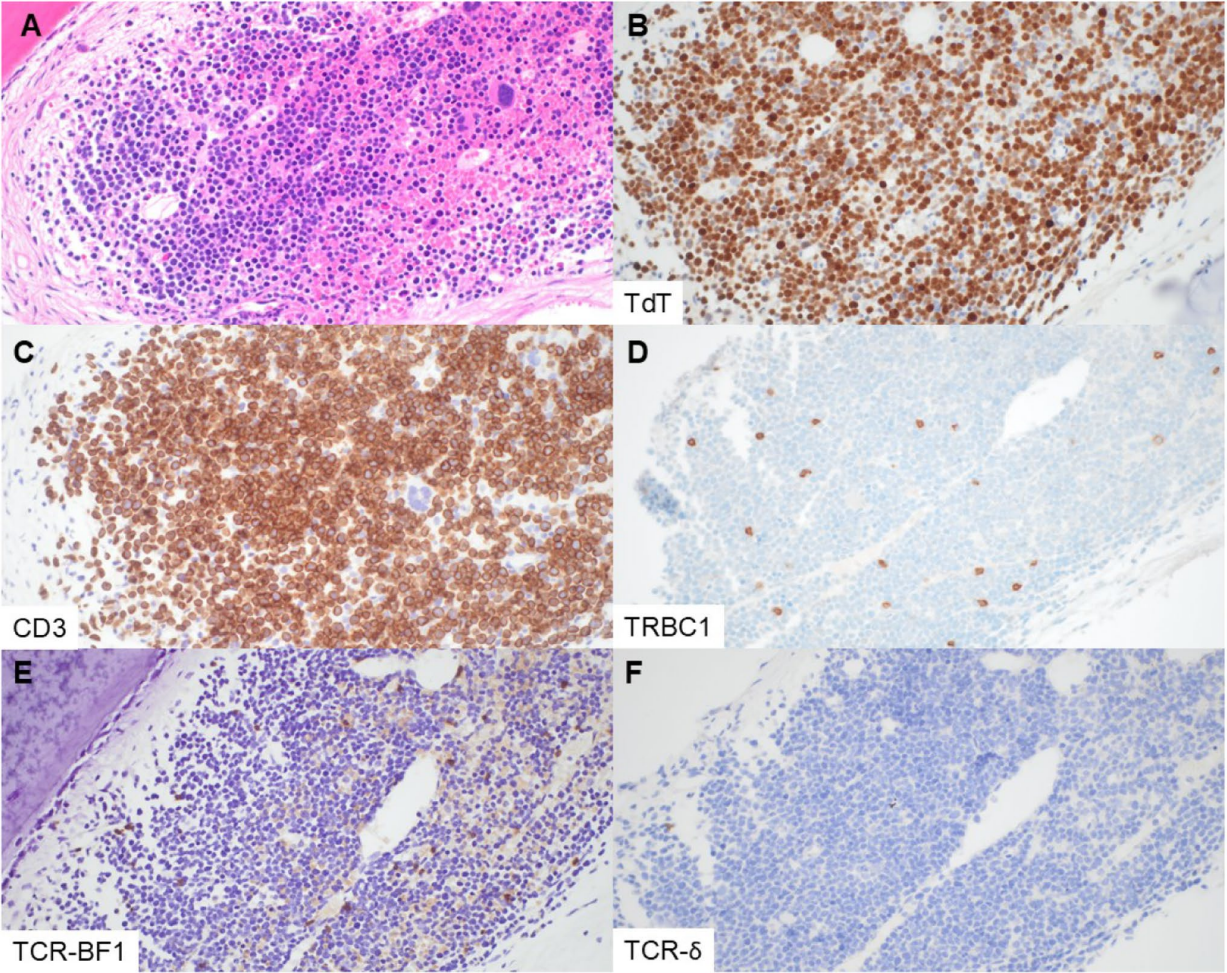
T-LL with negatively restricted TRBC1 expression and TCR null phenotype. This decalcified bone marrow core biopsy (patient 21, specimen 24) contains sheets of abnormal lymphoblasts (**A**, H&E), which are positive for TdT and CD3 (**B**, **C**). TRBC1 is negatively restricted in the blasts of interest, staining sparse scattered bystander T cells only (**D**). TCR-BF1 and TCR-δ are negative in the blasts (**E**, **F**) (magnification 400×, all panels)

**Fig. 5:**
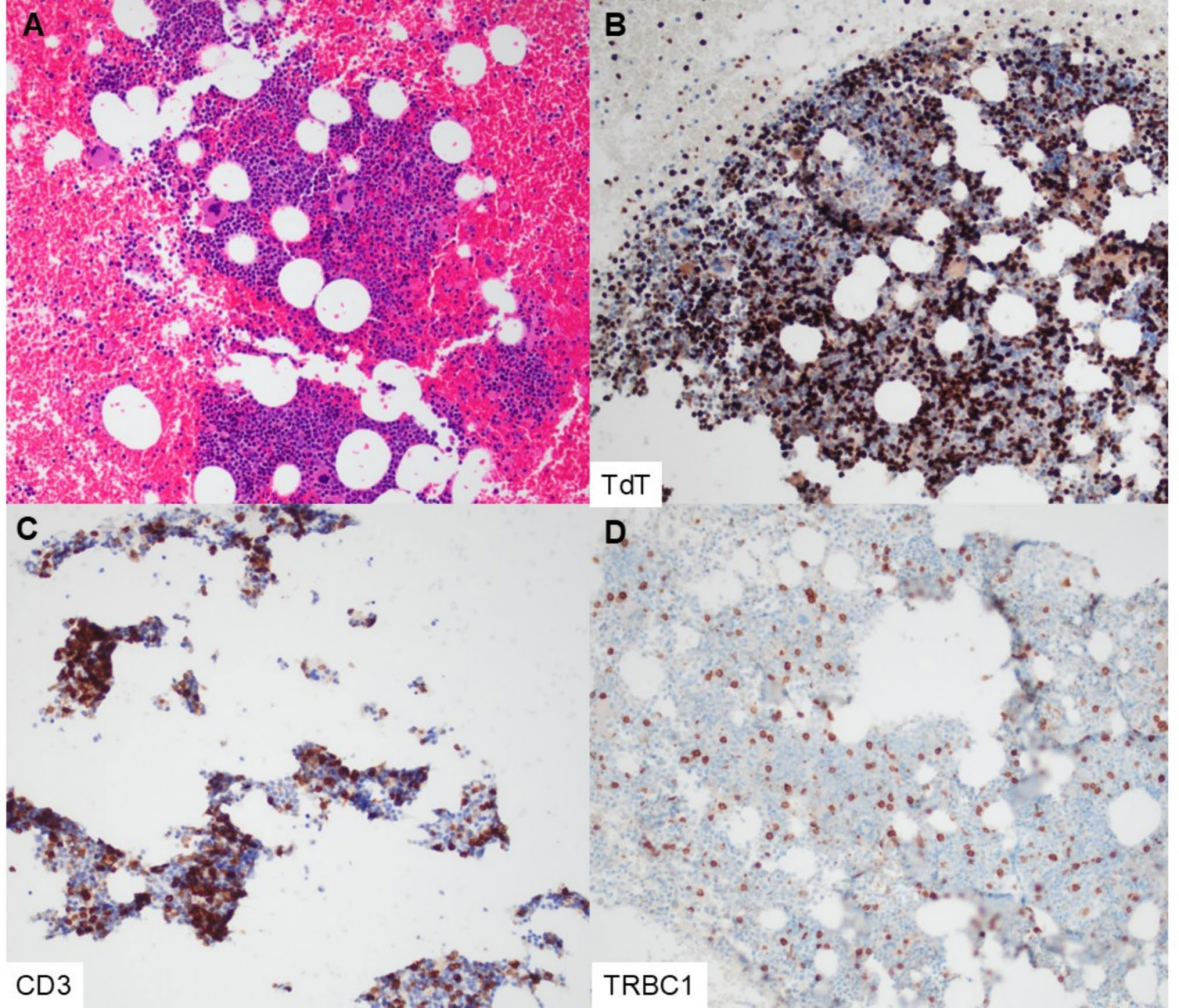
T-LL case requiring three-pathologist consensus for TRBC1 scoring. This bone marrow clot preparation (patient 3, specimen 4) demonstrates lymphoblasts that are distributed in a subtle, interstitial pattern amid a background of trilineage hematopoiesis (**A**, H&E). TdT is positive in the blasts and further highlights the interstitial distribution (**B**). CD3 appears positive in the blast population; however, interpretation is challenging due to the changing tissue profile, tissue loss/fragmentation, and the presence of background small T cells (**C**). TRBC1 is positive in scattered T-lineage cells but is far less prominent than CD3/TdT and was scored as negatively restricted by two of three pathologists (**D**) (magnification 200×, all panels)

All cases of T-LL showed detectable expression of CD3 and TdT in the lymphoblast population by IHC (Table 2), though the intensity (dim, moderate, strong) and degree (diffuse or partial) of staining varied across cases. All cases of T-LL were scored as having an abnormal pattern of TRBC1 expression by IHC. Three of 21 patients (14%), corresponding to 4 of 24 specimens (17%), showed a positively restricted TRBC1 staining pattern (Fig. 2). Eighteen of 21 patients (86%), 20 of 24 specimens (83%), showed a negatively restricted pattern (Figs. 3 and 4). The two reviewing pathologists showed concordant interpretations of TRBC1 IHC for 23 of 24 (96%) evaluated specimens. The one specimen that required three-pathologist review (patient 3, specimen 4, final consensus interpretation TRBC1 negatively restricted) was considered polytypic for TRBC1 in the blasts of interest by one of three pathologists. This was a case of partial bone marrow involvement, with an interstitial distribution of blasts, frequent admixed non-neoplastic T cells, and tissue loss/fragmentation (Fig. 5). For the three patients with two samples available for review (patients 2, 8, and 12), TRBC1 was scored consistently across both samples, including one patient (patient 2) with samples from initial diagnosis and relapse. In all cases, polytypic TRBC1 internal control staining was identified in background non-neoplastic T cells. Among five cases in which TRBC1 staining was performed on both the decalcified bone marrow core biopsy and bone marrow aspirate clot preparation, TRBC1 showed equivalent staining across sample types. Of the evaluated stains, TCR-BF1 most frequently required review by three pathologists for consensus (5 of 24 specimens, 21%). For patient 8, TCR-BF1 interpretation was discrepant across distant bodily sites, interpreted as weakly positive in bone marrow (TCR-BF1 +, specimen 9) and negative in lymph node (TCR-BF1−, specimen 10).

**Table 2:** Immunohistochemical and molecular features of T-lymphoblastic leukemia/lymphoma cases

TRBC1 and TCR category	Patient #	Specimen #	CD3	TdT	TCR-BF1	TCR-δ	TRBC1	TCR γ PCR	ClonoSEQ
TRBC1 + TCR-BF1 +	12	14	+	+	+ (VAR)	−	+		
	12	15	+	+	+	−	+	+	
	13	16	+	+	+ (W/VAR)	−	+	+	
TRBC1 + TCR-BF1-	5	6	+	+	−^c^	−	+	+	
TRBC1-TCR-BF1 +	1	1	+	+	+	−	−		+
	8	9	+	+	+ (W)	−	−		
	16	19	+	+ (W)	+	−	−	+	
	18	21	+	+	+ (W/VAR)	−	−	+	
	20	23	+	+	+	−	−	Failed to amplify	−
TRBC1- TCR δ +	2	2	+	+	−	+	−		
	2	3	+	+ (S)	−	+	−	+	
	4	5	+	+	−	+ (W/S)	−	+	
	6	7	+	+ (S)	−	+	−		
	7	8	+	+	−	+	−		
	9	11	+	+	−	+ (W/S)	−		+
	11	13	+	+	−	+ (W/S)	−	+	
TRBC1- TCR null	3	4	+ (W)^c^	+	−^c^	−	− ^c^		−
	8	10	+	+	−^c^	−	−	+	
	10	12	+	+ ^c^	−	−^c^	−		
	14	17	+	+	−^c^	−	−	+	
	15	18	+	+	−^c^	−	−		
	17	20	+	+ (S)	−	−	−		
	19	22	+ (W)	+	−	−	−	+	
	21	24	+	+	−	−	−		

*VAR* variable intensity staining in population of interest, *W* weak staining, *S* subset staining

C superscript designates cases requiring a third pathologist for consensus IHC interpretation

Positive and negative TRBC1 expression based on thresholds of 85% and 15%, respectively, in the T-lymphoblast population

**Fig. 6:**
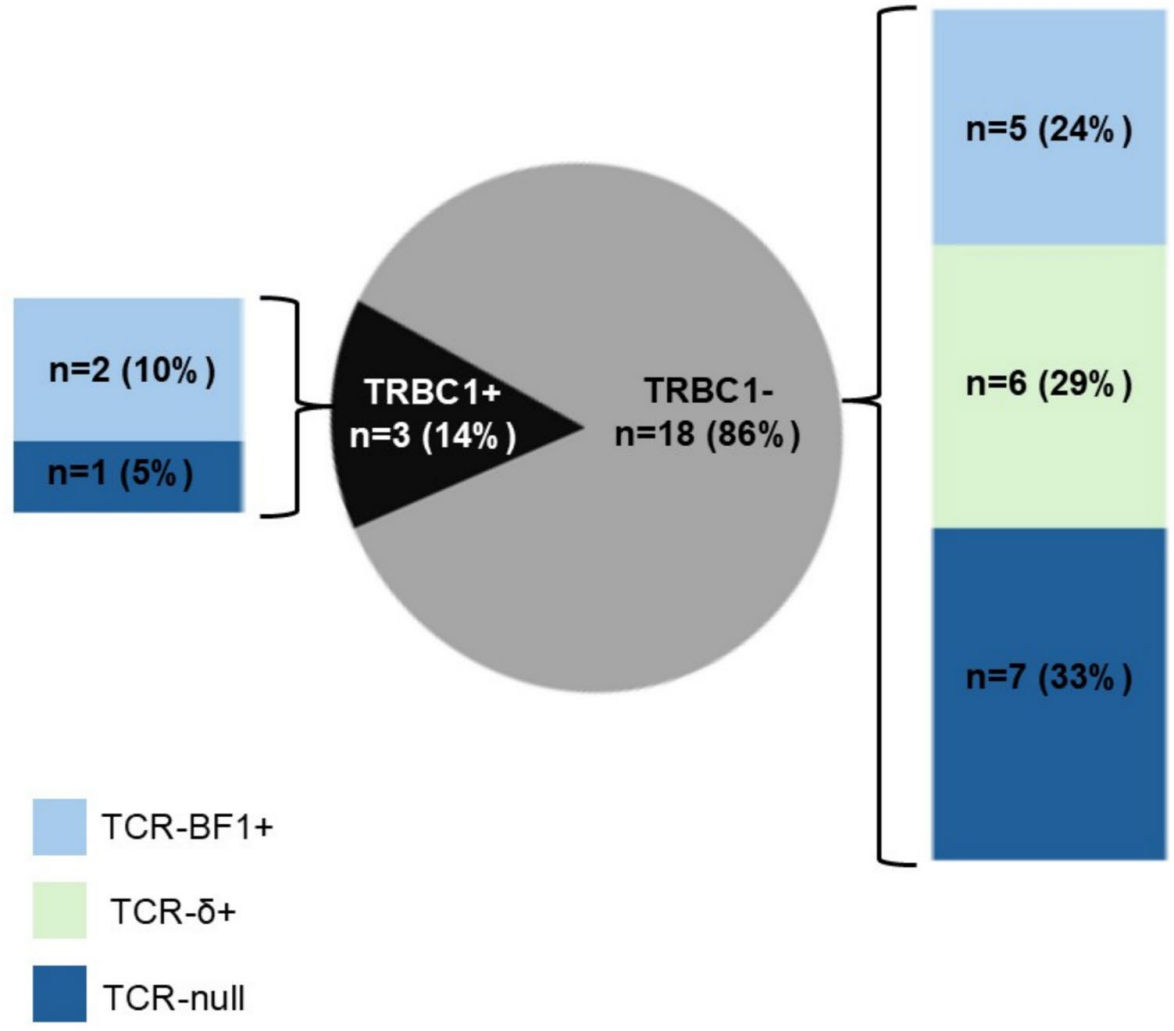
TRBC1 and TCR phenotypes in T-LL by Immunohistochemistry. As scored in 21 patients. *Patient 8: Scored as TCR-BF1 +, TRBC1− (specimen 9) and TCR null, TRBC1− (specimen 10). For purposes of the above schematic, patient 8 is included in the TCR-BF1 +, TRBC1− category

Further analysis of TRBC1, TCR-BF1, and TCR-δ staining revealed the following phenotypic profiles (summarized in Table 2 and Fig. 6):

1. TRBC1 positively restricted, TCR-BF1 positive, TCR-δ negative (2/21 patients, 10%).
2. TRBC1 positively restricted, TCR-BF1-negative, TCR-δ negative (1/21 patients, 5%). This case (patient 5) required a three-pathologist consensus review. Notably, TCR-BF1 was scored as negative by two pathologists; one pathologist commented on weak staining in a minor subset of blasts.
3. TRBC1 negatively restricted, TCR-BF1 positive, TCR-δ negative (5/21 patients, 24%). Patient 8 is included in this category due to the detection of TCR-BF1 staining in one specimen, suggestive of at least partial staining in the tumor cells.
4. TRBC1 negatively restricted, TCR-BF1 negative, TCR-δ positive (6/21 patients, 29%).
5. TRBC1 negatively restricted, TCR-BF1 negative, TCR-δ negative, (“TCR null,” 7/21 patients, 33%).

No clear trends were observed between TRBC1 staining (positive versus negative restriction) and the expression of additional immunohistochemical markers (CD1a, CD4, CD8, CD34, etc.), although the panels of markers evaluated were inconsistent across cases. Results of additional IHC markers scored in the T-lymphoblast populations of interest are included in Supplementary Table 1.

### Flow cytometry results

Concurrent flow cytometry data was available for 21 of 24 (88%) specimens, from 19 of 21 (90%) unique patients (Supplementary Table 2). One case (patient 3) had features suggestive of early T-precursor lymphoblastic leukemia/lymphoma (ETP-ALL), and one case (patient 9) had a near-ETP phenotype. The small number of cases and variable markers analyzed across cases precluded a formal statistical comparison of the IHC versus flow cytometry immunophenotypes. Regardless, no obvious relationships between TRBC1 IHC expression and the flow cytometry markers CD3 (surface and cytoplasmic), CD2, CD5, CD7, CD4, CD8, CD1a, CD34, and nTdT were apparent.

Three cases had TRBC1 expression evaluated by both IHC and flow cytometry (patients 3, 11, and 18), with all three uniformly negative for surface TRBC1 expression by flow cytometry and a negatively restricted TRBC1 expression pattern by IHC. Two of these cases demonstrated surface CD3 expression, and one lacked surface CD3 expression. Of the five patients (six specimens) in which surface TCR γ/δ was evaluated by flow cytometry, no cases showed surface TCR γ/δ expression, including one case (patient 11) that was positive for TCR-δ by IHC evaluation. Two patients had surface TCR α/β evaluated by flow cytometry. Patient 12 (specimens 14 and 15) showed TCR α/β expression (albeit dim) in a subset of the blast population of interest, concordant with the IHC finding of TCR-BF1 positivity. Patient 21 was negative for TCR α/β by flow cytometry and negative for TCR-BF1 by IHC.

### Molecular results

TCR γ gene rearrangement by PCR was performed on 12 patients (Table 2), with one patient (patient 20) having inadequate DNA amplification for analysis. The 11 patients with an interpretable study were all positive for a clonal rearrangement (100%).

ClonoSEQ results were available for four patients, of which two were positive for dominant clonal sequences (50%, patients 1 and 9) and two were negative for a dominant clonal sequence (50%, patients 3 and 20). Patient 3 was an ETP-ALL, with a TRBC1−, TCR null phenotype. Patient 20 was a TRBC1−, TCR-BF1 + T-LL with a CD4 + CD8 + (variable)CD1a + cortical thymocyte-like phenotype. As noted above, TCR γ gene rearrangement analysis was attempted on the specimen, but there was inadequate DNA amplification for interpretation.

Considering all TCR, PCR, and ClonoSEQ results together, molecular methods for clonality assessment were concordant with an abnormal TRBC1 IHC staining profile in 13 of 15 patients (87%).

## Discussion

In this study, we examined the expression of TRBC1 by IHC in immature T-cell proliferations. All cases of T-LL showed abnormal, “restricted” TRBC1 staining profiles, whereas benign thymic tissue and thymomas demonstrated a polytypic pattern of TRBC1 expression. Patients with multiple specimens showed consistent TRBC1 staining across tissue sites and sampling time points. TRBC1 IHC also showed excellent staining quality in decalcified bone marrow core biopsies. During the course of our study, a complementary IHC analysis of a similar cohort of immature T-cell populations was published by Lee et al. [17], describing non-restricted TRBC1 patterns in thymic tissue and restricted TRBC1 patterns in T-LLs (19% TRBC + and 81% TRBC1−). Our study corroborates their findings, identifying similar proportions of TRBC1 + (14%) and TRBC1− (86%) cases. As a novel feature, we also describe the phenotypic patterns of TRBC1 staining in relationship to TCR expression, with a large subset of cases showing TCR-δ + or TCR null phenotypes. Molecular methods for clonality assessment (TCR γ PCR, ClonoSEQ) were supportive of a clonal T-cell population in 13 of 15 (87%) tested cases, with the outliers being two cases lacking dominant clonal sequences by ClonoSEQ analysis. One case was an ETP-LL, which is known to have low rates of functional TCR gene rearrangement [18, 19]. The second discrepant case failed DNA quality controls at our institution, raising consideration for specimen quality limitations that may have impacted analysis.

Our study utilized both CD3 and TdT as comparator stains for TRBC1 evaluation in T-LL cases. While CD3 and TdT staining were positive in all cases, the intensity and degree of staining were not uniform, as expected based on the known phenotypic variability that can be seen in T-LL [20]. Accordingly, we found it helpful to have both immunophenotypic markers to delineate the extent and distribution of T-lymphoblast populations. Nevertheless, the TRBC1/CD3 and TRBC1/TdT scoring strategies each posed challenges. In addition to highlighting the immature populations of interest, CD3 stains background mature T cells, confounding evaluation in cases with frequent admixed non-neoplastic lymphocytes. Meanwhile, TdT was more specific for immature lymphoid populations, but tended to be more sensitive to fixation and decalcification effects in our laboratory. We selected cutoffs of ≤ 15% and ≥ 85% TRBC1 staining to define negatively and positively restricted cases, respectively. These thresholds have been repeatedly cited in flow cytometry literature [7–11] and have been employed to good effect in our daily flow cytometry practice. While practical, these cutoffs are admittedly arbitrary. The range of expected TRBC1 positivity shows mild variation across studies, based on the T-cell subsets and sample types analyzed [6, 8]. In our experience with T-LL cases, when the blast population is prominent and associated with tissue effacement, a formal quantitative assessment may not be necessary, as such blast populations are clearly uniformly positive or negative. Conversely, irrespective of scoring strategy, we anticipate that accurate TRBC1 IHC evaluation will be challenging (and in some instances impossible) in samples with low-level involvement by T-LL, particularly bone marrow. In this context, the blast population of interest will be admixed with frequent non-neoplastic cells, and comparator stains (CD3, TdT) are not entirely specific for the blast population of interest. This limitation was previously noted by Zhou et al. in their analysis of TRBC1 staining in mature T-cell lymphomas, in which TRBC1 IHC showed decreased sensitivity in the setting of low tumor burden [13]. In this regard, flow cytometry maintains an advantage over IHC methods, in its ability to detect small aberrant populations by evaluating TRBC expression in conjunction with additional immunophenotypic markers. To develop novel TRBC1% cutoffs for use in FFPE tissue, several groups have explored manual microscopy and digital analysis methods, with each reporting different optimal thresholds for defining positivity and negativity. Lower thresholds for TRBC1 positivity ranged from ≤ 25 to ≤ 36.3% while upper thresholds ranged from ≥ 70 to ≥ 79% [12–14, 17]. As our series is exclusively comprised of T-LL cases with high tumor burden and only contains three patients with positively restricted TRBC1, we feel it is not an ideal cohort to establish/validate diagnostic cutoff points.

As the frequencies of TCR and TRBC1 expression observed in our T-LL cohort differ substantially from those of mature T-cell lymphoproliferative disorders, further study of immature T-cell proliferations is warranted. Immunohistochemical assessment for TRBC2 would be an enticing next step, but unfortunately, a TRBC2 antibody suitable for IHC was not commercially available at the time of this study. Accordingly, for the 20 of 24 (83%) cases lacking TRBC1 expression, we could not determine the proportion of TRBC2-positive and TRBC null cases. We hypothesize that the TCR-BF1 +, TRBC1− subset of T-LL cases is the most likely subset to exhibit TRBC2 restriction. However, this should be further interrogated, particularly due to the challenges associated with TCR-BF1 IHC interpretation, which frequently required three-pathologist review (5/24 specimens, 21%) to distinguish dim/subset versus negative staining. This difficulty may stem from the innate biological variability of TCR expression in T-LL, in which expression can be dim, inconsistent across membranous and cytoplasmic cellular compartments, and/or limited to a subset of blasts [11, 21, 22]. In addition, we observed a large proportion of T-LL cases with apparent expression of TCR-δ (29%), and all TCR-δ + cases lacked TRBC1 expression. The significance of this finding is uncertain, as there is only limited data examining TCR-δ expression by IHC in T-LL cohorts. Normal maturing thymocytes show transient TCR-δ expression prior to maturation and adoption of a permanent TCR α/β phenotype [23, 24]. Furthermore, TCR-δ expression has been described in a subset of T-LL cases that lack both surface TCR γ/δ expression and mature γ/δ transcripts [25]. Thus, the mere expression of TCR-δ may not entirely correlate with a surface TCR γ/δ + phenotype, which is reported in only a small proportion of T-LLs (~ 9–12%) by flow cytometry [21, 26–29]. Indeed, for the one TCR-δ + case for which flow cytometry correlation could be performed (patient 11), surface TCR γ/δ expression was not observed.

The ability to correlate IHC staining with flow cytometric immunophenotyping was limited in this retrospective study, as the specific panels of evaluated flow cytometry markers varied across specimens. Surface TRBC1, TCR γ/δ, and/or TCR α/β assessments were included in only a minor subset of flow cytometry analyses, and cytoplasmic evaluation of these markers was not performed. Existing flow cytometry literature is also limited in this regard. The TRBC phenotype of T-LL has only been described for small case cohorts, with studies primarily reporting the profile of TRBC1, without concomitant assessment of TRBC2 [11, 15, 30, 31]. A paired analysis comparing the assessment of TRBC1, TRBC2, TCR α/β, and TCR γ/δ by immunohistochemical and flow cytometry methods (to include evaluation of surface and cytoplasmic expression) would be invaluable for establishing the spectrum of TCR/TRBC profiles in T-LL. TCR and TRBC transcriptional profiling is an additional technique that could be explored, but is beyond the scope of the current investigation.

While TRBC1 IHC is emerging as a useful tool for establishing clonality, a variety of morphologic, immunophenotypic, and molecular-cytogenetic features can be utilized to identify aberrant T-lymphoblast populations, and the primary diagnosis of T-LL is often straightforward when tissue sampling is adequate. However, TRBC1 IHC could be of added diagnostic value in select situations. For example, when evaluating specimens from the mediastinum, distinguishing between benign thymic hyperplasia, thymoma, and T-LL can be challenging, particularly in the setting of limited tissue and/or the absence of conclusive flow cytometric immunophenotyping. A limited IHC panel including CD3, TdT, TRBC1, and keratin may be sufficient to distinguish these entities, with the utilization of minimal tissue. Such a strategy would also be helpful for accurately differentiating ectopic thymic tissue from T-LL when evaluating small samples from the head and neck region. TRBC1 IHC could have utility in distinguishing indolent T-lymphoblastic proliferations from T-LL, as long as the lymphoblast population of interest is sufficiently prominent to be visualized/localized on serial tissue sections. Finally, the selective utilization of TRBC isoforms by T-cell neoplasms presents a compelling potential therapeutic target. An intervention that targets one TRBC isoform has selective activity against the neoplastic T-cell population of interest, while preserving sufficient background T cells to maintain host immunity [32–34]. In this setting, TRBC phenotyping by IHC may have utility in appropriately selecting patients for TRBC-targeted therapy.

In summary, TRBC1 expression assessment by IHC represents a viable alternative to flow cytometry for the evaluation of immature T-cell populations. T-LL cases with high tumor burden showed overtly restricted patterns of TRBC1 expression, whereas benign thymic tissue and thymomas consistently showed polytypic staining patterns. TRBC1 IHC may be particularly helpful in distinguishing between benign thymic tissue and T-LL when evaluating small samples from the mediastinum and head and neck. However, diagnostic utility is anticipated to be more limited in situations of low tumor burden. In this small series, TRBC1 IHC and molecular methods of clonality showed high concordance (87%), although TRBC1 was slightly more sensitive in identifying clonal populations. Our study demonstrates that patterns of TCR and TRBC1 expression are notably different in malignant T-lymphoblast populations compared to mature T-cell lymphomas, while highlighting the need to further refine the spectrum of TCR and TRBC expression in immature T-cell proliferations, with particular emphasis on exploring TRBC2 + and TRBC null phenotypes.

## Supplementary information

Below is the link to the electronic supplementary material.

ESM 1 (XLSX 21.5 KB)

## Data Availability

No datasets were generated or analysed during the current study.
